# Cancer risk among women with scoliosis: a nationwide danish register-based study

**DOI:** 10.1007/s43390-026-01301-5

**Published:** 2026-02-14

**Authors:** F. D. Højsager, L. Borch, R. Castelein, S. B. Christensen, A. Simony

**Affiliations:** 1 Department of Orthopaedic Surgery and Traumatology, Hospital Lillebaelt Kolding, Kolding, Denmark; 2 Department of Pediatric and Adolescent Medicine, Gødstrup Hospital, Herning, Denmark; 3 NIDO|Centre for Research and Education, Gødstrup Hospital, Herning, Denmark; 4 Department of Orthopedics, University Medical Center Utrecht, Utrecht, The Netherlands; 5 Department of Orthopaedic Surgery, University Hospital Rigshospitalet, Copenhagen, Denmark; 6 Department of Regional Health Research, University of Southern Denmark, Odense, Denmark; 7 Department of Orthopedic Surgery, The Region of Southern Denmark University Hospital, Sygehusvej 24, 6000 Kolding, Denmark

**Keywords:** Scoliosis, Cancer risk, Breast cancer, Register study

## Abstract

**Background:**

Adolescent Idiopathic Scoliosis (AIS) affects over 1% of adolescents and is often diagnosed during a sensitive developmental period, with repeated radiographs, potentially increasing cancer risk. Previous studies have suggested an association between scoliosis and breast cancer, but have been limited by small cohorts, lack of matched controls, and incomplete registry data. The aim of this study, therefore, was to assess the hazard ratio (HR) of cancer among women with scoliosis compared to age-matched controls using nationwide registry data.

**Methods:**

This observational cohort study included all Danish women born 1967–1977 with a scoliosis diagnosis (ICD-8 735.0/735.2 or ICD-10 DM411), and four age-matched controls per case, identified through national health registers. Cancer outcomes were obtained from the Danish National Cancer Register, and Cox regression was used to estimate HRs, with time at risk beginning at age 12.

**Results:**

Among 6,217 women (1,238 (20% with scoliosis), the mean follow-up time was approximately 38 years. The HR (95% CI) for any cancer was 1.06 [0.85–1.31]. For breast cancer, HR was 1.25 [0.86–1.80], while HR for carcinoma in situ (CIS) of the breast was 13.62 [3.75–49.50]. Among 12 cases of ovarian cancer, the HR was 2.91 [0.92–9.18]. This study could not, however, discriminate between idiopathic and non-idiopathic scoliosis.

**Conclusion:**

Women with scoliosis showed a significantly increased HR for CIS of the breast, and a non-significant trend toward increased risk of invasive breast and gynecologic cancers. Findings support further investigation into genetic or developmental links between scoliosis and cancer risk.

**Key Points:**

- Women with scoliosis are at a higher risk of getting cancer, namely of the breast.
- Pregnancy did not appear to have a protective effect.

## Introduction

Adolescent Idiopathic Scoliosis (AIS) has a prevalence of 4–5% and is the most common form of scoliosis [1]. In AIS rotational stability in the spine is compromised, resulting in a three-dimensional (3D) s-shape of the spine, due to biomechanical alterations during the final growth spurt [2]. While the etiology of AIS has not yet been established, association with genetic factors have been identified [3, 4].

Scoliosis is diagnosed by clinical examination, eg. Adams forward bending test [5], use of scoliometer [6], and by use of radiographs of the spine in both Posterior-Anterior (PA), and Lateral projections.

When undergoing radiographs, a dose of ionizing radiation is absorbed in the body, varying by tissue and projection [7]. The dose of radiation towards the breast in an AP projection of the spine, has been reduced drastically throughout the twentieth century, with modern techniques exposing the patients to 1/6th of the radiation used on children below 13 years of age in 1940–1959, from 0.780 centigray to 0.125 centigray [8]. The current golden standard is to perform PA projections, which have a low radiation of 0.003 centigray towards the breast tissue [8].

Associations between risk of cancer and exposure to ionizing radiation are well documented [9–11], and former American studies have identified an excess relative risk of cancer of 5.4/gray for women with scoliosis [8]. Several studies have previously estimated whether women with scoliosis were more prone to being diagnosed or dying of cancer, compared to non-scoliotic women [8, 12–14]. The main focus has been on breast [12, 14] or endometrial cancer [14] as it has previously been assessed whether there is an increase in cancer rate among AIS patients [14]. The most predominant focus has been on breast cancer, due to scoliosis often being diagnosed from age 12, which is also the period for breast and pelvic organ maturation and growth [15], and due to historical data from nuclear bomb survivors, stating breast tissue was the most sensitive [16]. Both breast and pelvic organs are exposed during spine radiographs. Candidate gene studies searching for the cause for scoliosis has reported on a variety of different candidate genes eg. VANGL1 or LBX1 [17–19], VANGL1 is also known as a gene related to cancer [18].

No previous study has reported on a nationwide cohort and examined the probability of cancer in women with scoliosis, compared to age-matched healthy controls, while taking competing risk factors into account. The aim of this study was to estimate the hazard ratio of cancer among women with scoliosis compared to women without scoliosis, focusing on previously addressed cancers, while correcting for death and emigration as competing factors.

## Methods

### Data sources

This observational cohort study was based on data obtained from the Danish National Health registers. Patients were sampled according to registrations used in the Danish National Patient Register (DNPR). The DNPR has a completeness rate of > 99% and has collected data from all Danish somatic hospitals since 1977 [20]. The Danish National Cancer Register (DNCR) was used for the inclusion of all registered diagnoses. The DNCR contains records of all cancers in the Danish population from 1943, with reporting being mandatory from 1987 [21–23]. The DNCR contains data on all malignant diseases as well as a select group of non-malignant tumors [24]. For linking included patients between the registers, an encrypted personal identification number (PIN) from the Danish Civil Registration System (DCRS) was used [25]. Data on whether the person was alive and living in Denmark were obtained from the DCRS as well, as they are updated daily on all persons with a Danish PIN [25]. The Danish Birth Register (DBR) was used to address whether the women were registered as mothers [26].

### Sampling strategy

The cohort consisted of all women born between 1967 and 1977, who were registered in the DNPR with ICD-8 codes 735.0 (Scoliosis) or 735.2 (Kyphoscoliosis) or with the ICD-10 code DM411 (Adolescent Idiopathic Scoliosis). Furthermore, as controls, the cohort consisted of 4 age-matched healthy women for each woman with scoliosis. No woman served as a control for more than one woman with scoliosis.

### Data management and statistics

The data was provided by the Danish Health Data Authority using a remote desktop solution. In the data environment, a total of 6 data frames were provided, which were cleaned and merged using R version 4.3.3 in RStudio 2024.12.1 Build 563. Statistics were performed on the resulting dataset using STATA/MP 18.5.

Baseline characteristics were reported based on data available in the DCRS, DBR, and DNCR databases. Although risk time did not strictly follow a normal distribution, it was reported as mean and SD to ease comparison to other studies.

The hazard of being diagnosed with cancer was assessed using a Cox Regression. The time of origin was set to the birthday of the patient, while exit was either at the time of diagnosis or the time of censoring by death or emigration, thereby correcting for death as a competing factor of being diagnosed with cancer. Assuming the patients needed to be examined and radiographed for scoliosis prior to cancer diagnosis, risk time was defined as starting from the patients’ 12th birthday. Patients with a cancer diagnosis prior to age 12 and patients who were censored due to death or emigration, prior to age 12 were excluded from the statistical analysis. ICD code C44 were not included in the calculation of HR of any cancer, as it is the standard code for reporting Basal Cell Carcinoma, which is not considered malignant. If a person was registered multiple times in the DNCR within the range of ICD-codes defined as cancer, only the time to the first diagnosis was used in the analysis, as a person would otherwise contribute with more than one event, and observations therefore would not be independent.

A biostatistician was consulted regarding choices of modelling and model assumptions. He further performed a code review of the pseudocode.

Hazard ratios were presented for cancers identified in other studies, as well as for any cancer registration in the DNCR.

## Results

**Fig. 1:**
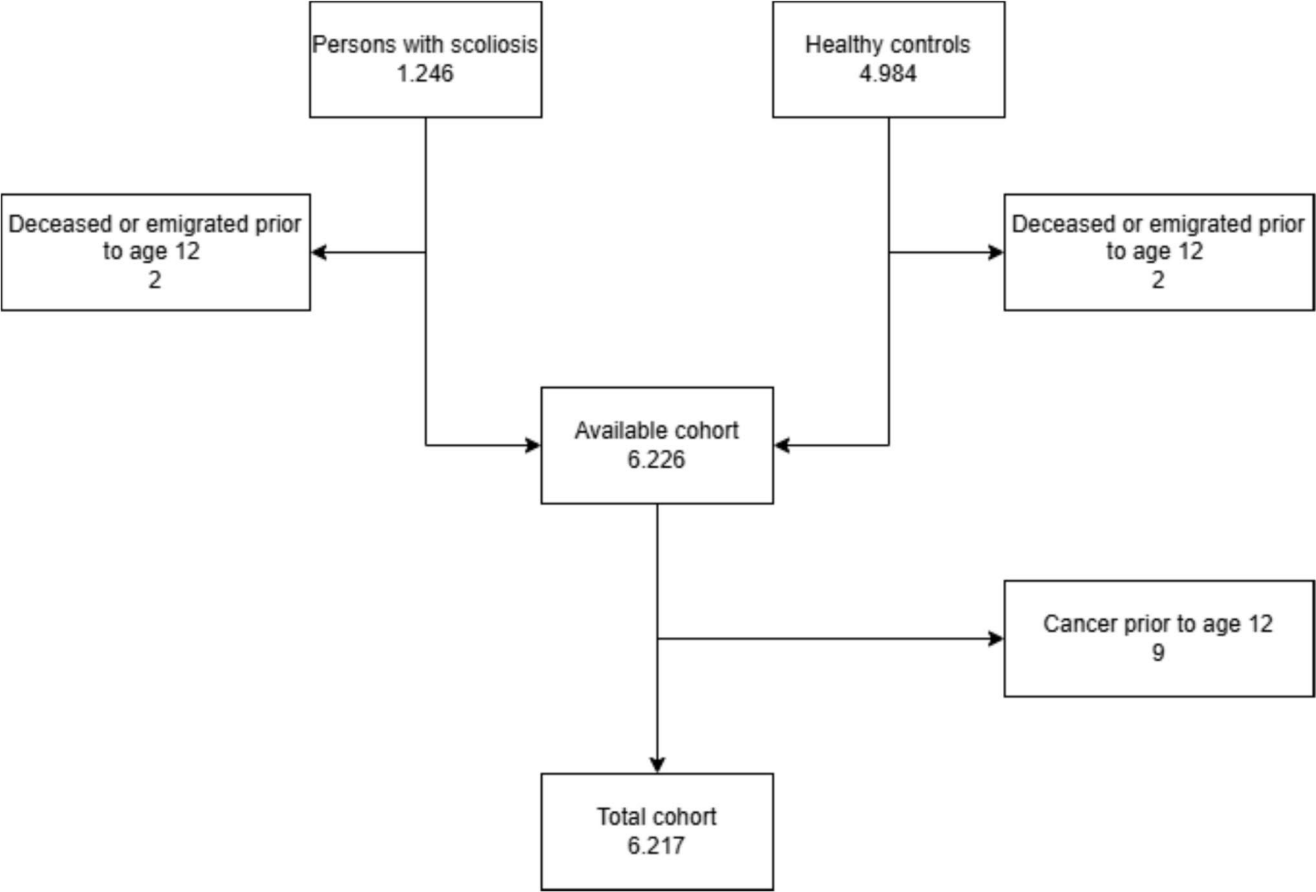
Flowchart showing included persons in the cohort. Women who are deceased, emigrated or diagnosed with cancer prior to age 12 are excluded

The study included a total of 6.230 women, of which 20% (*n*_*s*_ = 1.246) were diagnosed with scoliosis. Of the included women, 4 were either deceased or had emigrated prior to age 12, rendering an available cohort of 6.226 women (*n*_*s*_ = 1.244). A further 9 women were excluded due to a registration of cancer prior to age 12, yielding a final cohort of 6.217 women (*n*_*s*_ = 1.238) (Fig. 1).

### Characteristics

In the analysis of the hazard of any registration with cancer, the cohort contributed a total of 232.391 person-years at risk. The mean risk rime (SD) contributed by women with scoliosis was 37.85 (6.23), with non-scoliotic women contributing with a mean of 37.91 (5.38) years at risk (Table 1) corresponding to a mean age in the cohort of roughly 50 years. Of the women with scoliosis, 8.8% were censored due to dying or emigrating during the study period, with 6.2% of the controls being censored.

**Table 1:** Table of baseline characteristics of women included in the cohort

	Scoliosis (*n* = 1.238)	Non-scoliosis (*n* = 4.979)
Risk time^a^ (mean, SD), years	37.85 (6.23)	37.91 (5.38)
Died or emigrated during study period (n, %)	109 (8.8%)	308 (6.2%)
Mothers (n, %)	284 (22.9%)	1.196 (24.0%)
Age at first registration in cancer register (mean, SD), years	36.91 (9.19)	36.89 (8.79)

^a^Risk time starts at age 12 years

### Hazard of cancer

When accounting for death and emigration as competing factors, the overall crude HR [CI] of being diagnosed with any neoplasm, among 714 cases, was 1.11 [0.93;1.32] for women with scoliosis compared to women without scoliosis (Table 2). Adjusting for maternal status did not alter this estimate. When constraining to any cancer diagnosis, apart from DC44x, the overall crude HR [CI] was 1.06 [0.85;1.31], among 501 cancer cases. Formerly identified cancers of the breast, endometrium, or ovaries were estimated with a crude HR [CI] of 1.25 [0.86;1.80] for 159 breast cancer cases, 2.74 [0.46;16.41] for 5 endometrium cancer cases, and 2.91 [0.92;9.18] for 12 ovarian cancer cases (Table 2). Of 12 women with ovarian cancer, one of them was also registered with breast cancer. In estimation of Carcinoma in Situ (CIS) of the breast, overall crude HR [CI] was estimated at 13.62 [3.75;49.50] for 13 cases of CIS of the breast (Table 2).

**Table 2:** Hazard rates of cancers previously described in literature, as well as all cancers or neoplasms

Diagnosis	ICD10 code	N_cancer_	N_scoloisis_	N_control_	HR_crude_ [CI]	P_crude_	Hr_adjusted_ [CI]	P_adjusted_
Neoplasm	C0-D48	714	152 (12.3%)	562 (11.3%)	1.11 [0.93;1.32]	0.27	1.11 [0.93;1.32]	0.27
All cancers	C0-D0xx (not C44)	501	103 (8.3%)	398 (8.0%)	1.06 [0.85;1.31]	0.63	1.06 [0.85;1.31]	0.63
Melanoma	C43x	87	13 (1.1%)	74 (1.5%)	0.72 [0.40;1.29]	0.27	0.72 [0.40;1.29]	0.27
Breast cancer	C50x	159	37 (3.0%)	122 (2.5%)	1.25 [0.86;1.80]	0.24	1.25 [0.86;1.80]	0.24
Endometrial cancer	C54x	5	2 (0.2%)	3 (0.1%)	2.74 [0.46;16.41]	0.27	2.75 [0.46;16.44]	0.27
Ovarian cancer	C56x	12	5 (0.4%)	7 (0.1%)	2.91 [0.92;9.18]	0.07	2.91 [0.92;9.18]	0.07
Thyroid cancer	C73x	17	4 (0.3%)	13 (0.3%)	1.26 [0.41;3.85]	0.69	1.26 [0.41;3.85]	0.69
Carcinoma in situ of the breast	D05	13	10 (0.8%)	3 (< 0.1%)	13.62 [3.75;49.50]	< 0.001	13.55 [3.73;49.24]	< 0.001
Carcinoma in situ of the cervix	D06	122	26 (2.1%)	96 (1.9%)	1.10 [0.71;1.70]	0.66	1.10 [0.71;1.70]	0.66

Adjusted for maternal status

In the analysis of thyroid cancer, the crude HR [CI] was found to be 1.26 [0.41;3.85] in 17 cases.

For melanomas however, the crude HR [CI] was 0.72 [0.40;1.29] (Table 2).

Estimates of cancers, not previously addressed in published literature are shown in Table 3. A majority of these had less than 10 total cases, with several only having cancer cases in the control group, rendering HR impossible to calculate. Both Hodgkin and non-hodgkin lymphomas had a crude HR of 2.04, although only 3 cases of each. In estimation of 32 gynoid cancers, not formerly being addressed in papers, a crude HR [CI] of 0.42 [0.13;1.38] was seen, with a vast majority of these being cervical cancer.

**Table 3:** Hazard rates of cancers seldom described in literature or cancers with few cases

Diagnosis	ICD10 code	N_cancer_	N_scoloisis_	N_control_	HR_crude_ [CI]	P_crude_	Hr_adjusted_ [CI]	P_adjusted_
Oral cancers	C0xx-C14x	6	1 (0.1%)	5 (0.1%)	0.81 [0.09;6.95]	0.85	0.81 [0.09;6.94]	0.85
Digestive cancers	C15x-C26x	32	6 (0.5%)	26 (0.5%)	0.94 [0.39;2.29]	0.90	0.94 [0.39;2.28]	0.89
Thoracal cancers	C3x	12	2 (0.2%)	10 (0.2%)	0.82 [0.18;3.73]	0.79	0.82 [0.18;3.73]	0.79
Bone or cartilagenous cancers	C41x-C42x	1	0	1 (0.1%)				
Non-breast, non-endometrial, non-ovarian gynoid cancers	C51x-C53x & C55x & C57x-C59x	32	3 (0.2%)	29 (0.6%)	0.42 [0.13;1.38]	0.15	0.42 [0.13;1.38]	0.15
Urinary tract cancers	C64x-C68x	4	0	4				
Cancer of the eye	C69x	1	0	1				
Cancer of the brain or CNS	C70x-C72x	6	0	6				
Adrenal cancer	C74x	0	0	0				
Hodgkin lymfoma	C81x	3	1 (0.1%)	2 (< 0.1%)	2.04 [0.19;22.55]	0.56	2.07 [0.19;22.84]	0.55
Non-hodgkin lymfoma	C82x-C86x	3	1 (0.1%)	2 (< 0.1%)	2.04 [0.18;22.47]	0.56	2.03 [0.18;22.37]	0.56
Leukaemia	C91x-C95x	7	1 (0.1%)	6 (0.1%)	0.68 [0.08;5.62]	0.72	0.68 [0.08;5.68]	0.73

Adjusted for maternal status

**Fig. 2:**
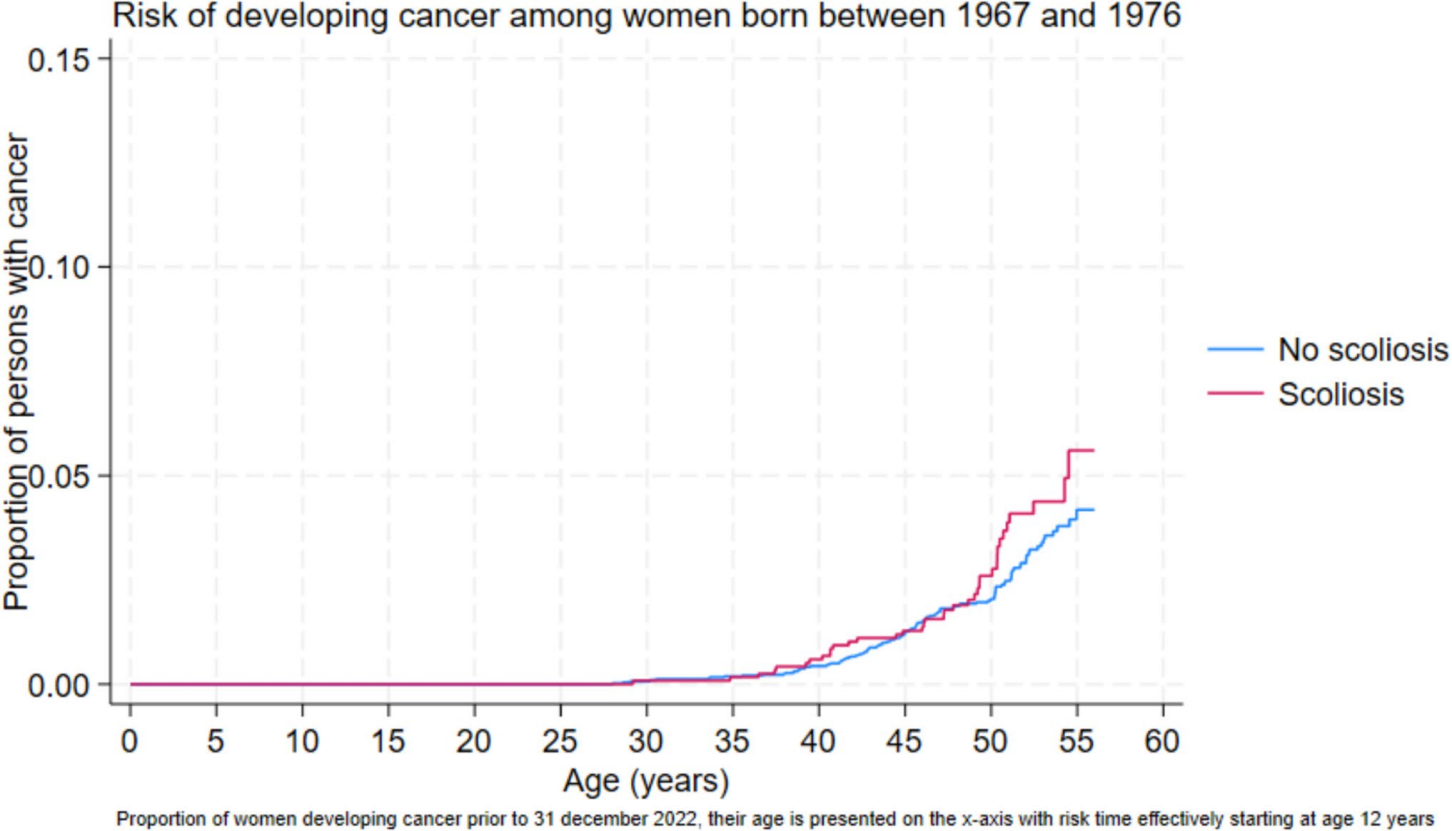
Kaplan–Meier curve of proportion of 6217 included persons diagnosed with breast cancer. Time at risk is defined from age 12. Persons are censored at death, emigration and exit at diagnosis. The included women are born between 1967 and 1976

As breast cancer was the most common cancer, a Kaplan–Meier curve was made. In the plot, the difference between groups seems to be apparent starting around age 50 and continues to be present throughout the follow-up (Fig. 2).

## Discussion

In this large register study, we investigated the HR of several cancers in women with scoliosis compared to women without scoliosis. The HR of any neoplasm was 1.11, the HR of breast cancer was 1.25, while the HR of CIS of the breast was 13.62. While the HR for neoplasms and breast cancer were not significant, the HR for CIS was highly significant with *p* < 0.001, with an age range of 29.4–54.9 years at the time of diagnosis of CIS.

To the best of our knowledge, this is the largest modern study of risk of cancer in women with scoliosis, the first addressing carcinoma in situ, and the largest using central mandatory cancer registries.

Historical cohorts tie an excess risk of cancer among women with scoliosis to their exposure to ionizing radiation [27]. However, other potential comorbid, genetic, or epigenetic factors associated with spinal imbalance may also influence cancer risk [17–19].

Our findings are in accordance with data from the American Scoliosis Cohort, where Hoffman et al. found a standardized incidence ratio (SIR) for breast cancer of 1.8 [12] for women with scoliosis compared to the national average. The difference could be attributed to a decrease in radiation in ionizing radiation [8]. Other studies from the American Scoliosis Cohort, further identified increased cancer mortality among women with scoliosis [8, 28]. The mean age of the American cohort was 51 [8], compared to our approximately 50 years, although they had a much larger age spread than the women in the present study. A recent Australian study [13] conversely found a SIR of 0.83 for any type of cancer. Although this opposes our findings, the reason could be, their follow-up time being relatively low at 18 years, compared to our approximately 38 years at risk which is still low for a thorough evaluation of the development of breast cancer. In a Dutch study, Heijboer et al. found a standardized prevalence ratio of 1.10 for any non-BCC cancer, among women with a mean age of 44 years. Their study design was akin to ours, and their findings are in accordance with ours; they did, however, not compare to matched controls but compared to the general Dutch female population. Furthermore, they stressed the possibility, that they could be underpowered, with 337 women with scoliosis. Lastly a previous observational study by Simony et al. [14] reports a relative risk of cancer of 4.8 in AIS patients, compared to population data from the NORDCAN database [29] in women with scoliosis and a mean age of 37.6 (range 34.5–47.0) years. The findings were based on a very small cohort of 9 cancer cases, possibly overestimating the risk, due to the study design and limited number of patients included [30]. None of the former studies addressed death, emigration or other reasons for censoring as competing events, nor did they try to factor in pregnancy as a protective factor.

This study has several implications. The most notable implication is that, although non-significant, we found the same trends of increase in breast cancer among women with scoliosis, as has previously been described in the American scoliosis cohort [8, 12, 28, 31], as well as more recent Australian [13], Dutch [32], and Danish studies [14]. With a mean age of approximately 50 in our cohort, this is also on par with the other cohorts, emphasizing the importance of our findings. Furthermore, the large HR of CIS could be considered a precursor for risk of breast cancer, although the clinical importance of CIS as a breast cancer precursor is debated [33]. Curiously, we identified an increase in hazard even though the radiation dose towards the breast has been reduced remarkably from the period of the American studies, and to the diagnostic period of our cohort [8]. Furthermore, as patients involved in this cohort are most likely to have been diagnosed with PA projections, the impact of ionizing radiation on cancer of the breast should be negligible. This could also imply that there might be predispositions to cancers linked to possible scoliosis genes eg. VANGL1, LBX1 [17–19], which could be a subject for future research.

Some limitations need to be noted. Firstly, our study cannot discriminate between idiopathic and non-idiopathic scoliosis, as the DNPR used ICD-8 up until 1994 [34]. This could be accommodated by limiting the cohort to women registered with the ICD-10 code for AIS. This would, however, limit the cohort to women having a hospital contact after age 20 with AIS, rendering a substantial selection bias. Using the wider ICD-8 code for scoliosis diminishes selection bias, at the cost of this only being generalizable to women with different types of scoliosis and not exclusively AIS.

Secondly, the mean age of women included was around 50 years. The consequence is that this study only identifies cancers that are early or relatively early onset. Since the major difference in breast cancer starts to become apparent from age 50, and our follow-up stops a few years later, repeat studies should elucidate whether the apparent trend identified in this study continues to become more obvious with longer follow-up.

Due to limitations in the registries, we could not identify exact doses of radiation, nor could we find the exact number of radiographs the women had received.

Furthermore, this study could not account for smoking or sexual history, which are strong risk factors for several cancers.

Although a variable for whether the women were identified as mothers in the DBR was made, the proportion appeared low, with around 1 in 4 being mothers, which could be due to missingness in the variable. As such, the adjusted HR were not considered our main findings.

Lastly, for some cancers, although the HR was > 2, the number of cases was modest. Therefore, the corresponding confidence intervals were extremely broad, with the risk of overestimating the effect even in the statistically significant ones [30]. In the interpretation of e.g. the HR of CIS of the breast caution is warranted, as the true HR could very well be lower than our estimates in studies with more cases.

These results support the notion that scoliosis could be linked to an increased risk of cancer. Although this is the largest study, with modern standards of radiography and therefore less exposure to ionizing radiation, this warrants repeat analysis of hazard ratio in other, older cohorts, where data validity is akin to the Danish registers.

## Conclusion

This nationwide cohort study found a non-significant increase in overall cancer and breast cancer probability among women with scoliosis, and a significantly higher risk of carcinoma in situ of the breast. Despite reduced radiation exposure in modern diagnostics, the findings suggest a potential link between scoliosis and cancer risk, possibly due to underlying genetic factors. Further research is needed to confirm these associations and assess long-term outcomes.

## Data Availability

The data that support the findings of this study are provided by the Danish Data Protection Agency. Restrictions apply to the availability of these data, which were used under license for this study.
